# A two-week, double-blind, placebo-controlled trial of *Viola odorata, Echium amoenum* and *Physalis alkekengi* mixture in symptomatic benign prostate hyperplasia (BPH) men

**DOI:** 10.1080/13880209.2017.1328445

**Published:** 2017-05-23

**Authors:** Fatemeh Beiraghdar, Behzad Einollahi, Alireza Ghadyani, Yunes Panahi, Abbas Hadjiakhoondi, Mahdi Vazirian, Ali Salarytabar, Behrad Darvishi

**Affiliations:** a Nephrology and Urology Research Center, Baghiatallah University of Medical Sciences, Tehran, Iran;; b Department of Pharmacognosy and Medical Plants Research Center Faculty of Pharmacy, Tehran University of Medical Sciences, Tehran, Iran;; c Integrative Oncology Department, Breast Cancer Research Center ACECR, Iran;; d Recombinant Proteins Department, Breast Cancer Research Center ACECR, Tehran, Iran

**Keywords:** IPSS score, quality of life, prostate volume, extant urine volume

## Abstract

**Context:** As an alternative approach, administration of phytotherapeutic agents in management of benign prostate hyperplasia (BPH), is rapidly growing each day. Different authors have indicated effectiveness of *Viola odorata* L. (Violaceae), *Echium amoenum* Fisch. & C.A.Mey. (Boraginaceae) and *Physalis alkekengi* L. (Solanaceae) in treatment of BPH. However, none have reported the beneficial outcomes of the mixture yet.

**Objective:** This study evaluates the therapeutical effects of *V. odorata*, *E. amoenum* and *P. alkekengi* mixture on symptomatic BPH patients.

**Materials and methods:** Eighty six symptomatic BPH patients with International Prostate Symptom Score (IPSS) of more than 13 and prostate volume of more than 30 cm^3^ were randomly allocated to receive a two-week course of placebo (control group) or 1 mL of mixed hydro-alcoholic solution of *P. alkekengi*, *E. amoenum* and *V. odorata* extracts (1.5, 1 and 1.5% respectively) (treatment group).

**Results:** IPSS score of incomplete urination (42.3 ± 2.04%), frequency of urination (20.08 ± 1.02%), intermittency (40.78 ± 2.16%), urgency (60.91 ± 3.14%), weak stream (50.58 ± 2.14%), straining (55.67 ± 2.53%) and nocturia (40.14 ± 1.89%) in treatment group were significantly decreased after treatment compare to placebo receiving group. Furthermore, the prostate volume (16.92 ± 0.89%) and extant urine volume (28.12 ± 1.36%) also significantly decreased in treatment group compared to control group. No significant side effects or abnormalities in biochemical tests and urinalysis were observed throughout the study.

**Discussion and conclusions:** Based on results, mentioned mixture is safe and effective in improving life quality of patients suffering from BPH.

## Introduction

Benign prostatic hyperplasia (BPH) is considered as the most prevalent urologic disorder in elderly men with prevalence rate of more than 70% at 60 years old age and 90% older than 70 years. It is mostly diagnosed by stromal and glandular cells hyperproliferation around periurethral areas and transition zones of the prostate gland (Untergasser et al. 2005; Chughtai et al. 2011; Bostanci et al. 2013). Lower urinary tract symptoms (LUTS) are the most prevalent complaints associated with this disorder (Untergasser et al. 2005; Nickel 2008). Initiation of hyperplasia in transition zone causes resistance in urinary outflow which in turn, eventually results in development of detrusor dysfunction, bladder trabeculation, and uninhibited bladder contractions (Aaron et al. 2016; Kim et al. 2016). In more severe stages, untreated BPH will lead in complications such as urinary tract infection, acute urinary retention and ultimately, obstructive nephropathy (Alivizatos & Skolarikos 2008). Although in most cases initiation of treatment relieves most of BPH symptoms, still urinary tract obstruction can cause major health problems such as bleeding from the prostate, recurrent infections, bladder stones, inability to urinate, kidney insufficiency or failure (Tewari et al. 2013). Therefore, it is essential to identify and initiate effective treatment strategies in order to overcome these complicated situations in BPH patients.

Despite the diversity of theories describing the progressive nature of hyperplastic processes involved in etiology of BPH, the certain pathogenesis has not been fully understood yet. Initially, it was assumed that an increase in dihydrotestosterone concentration, the most potent androgen motivating differentiation and growth in adult male, is the key point in development of BPH (Andriole et al. 2004). Today, although this hypothesis has been proven to be incorrect, as the concentration of DHT is eventually decreased with age in elderly men, still 5α-reductase inhibitors are continued to be prescribed with limited success. Additional evidence against DHT hypothesis came from the finding that DHT is only involved in differentiation but not the proliferation in the prostate gland. Now, as an established fact, this is the increase in ratio between prostatic estradiol and DHT in aging prostate gland which causes imbalancement in endocrine homeostasis (Schalken 2015). Recently, accumulating evidence suggests that inflammation is the key motivator of prostatic hyperplasia progression (Bostanci et al. 2013).

Currently, diverse medication regimens exist for relieving symptoms, slowing the growth of prostate and decreasing the development of future urinary complications in men suffering from LUTS due to BPH (Narayan & Tunuguntla 2005), which can be basically classified into three groups: α-blockers (e.g., Terazosin, Doxazosin and Tamsulosin), 5-α-reductase inhibitors (e.g., Finasteride and Dutasteride) and alternative therapies including changes in life style and minimally invasive therapies (Tarter & Vaughan 2006; Miller & Tarter 2009; Shrivastava & Gupta 2012). Newly developed procedures have made medication process so easy that even some of obstruction relieving interventions can be performed in the urologist’s office or as an outpatient procedure. Also, old-style surgical therapies for BPH such as transurethral resection of the prostate (TURP) can be performed more safely, shorter hospital bedridden, quicker recovery and fewer surgical and post-surgical problems than before (Clark et al. 2004; Bullock & Andriole 2006). Nevertheless, clinicians must be aware of cases in which the patients are allergic to these medications. In addition, surgical methods may also affect the quality of life. Furthermore, patients with BPH are also prone to acquire drug-related problems (DRPs), defined as events associated with drug therapy that actually or potentially impede with favoured health outcomes. Regarding therapy in patients with BPH, these include adverse reactions, administration of inappropriate drug of choice, dose adjustment problems, polydrug therapy and multiple comorbidity associated problems (Huri et al. 2014).

As an alternative therapy in BPH, phytotherapy or administering plant extracts with therapeutic purposes to manage BPH, is rapidly growing each day (Keehn et al. 2016). Administration of phytotherapeutic agents for treatment of BPH is rapidly growing in Europe. In Germany, mild to moderate urinary obstructive symptoms are mainly treated with phytomedicines and represent more than 90% of all drugs administered to treat patients with BPH (Zegarra et al. 2007). Phytomedicines are also readily consumed as nonprescription dietary supplements in USA and are frequently recommended in ‘natural health-food’ stores for self-treating BPH symptoms (Keehn & Lowe 2015). So far, about 30 phytotherapeutic agents have been identified in treatment of BPH and the number is growing each day (Macdonald et al. 2012). *Viola odorata* L. (Violaceae), *Echium amoenum* Fisch. & C.A.Mey. (Boraginaceae) and *Physalis alkekengi* L. (Solanaceae) are three of these agents applied in current study.

*Physalis alkekengi*, also referred as ground cherry, is an indigenous herb in Iran and many other regions of Asia such as China. Studies have reported presence of several active compounds including physalins, alkaloids, flavonoids and megastigmane glycosides in *P. alkekengi* (Qiu et al. 2008). A growing body of evidence exists that *P. alkekengi* demonstrates several therapeutic activities against various kidney and urinary disorders, soothing and diuretic effects, and more importantly, controls urine discharge, bleeding and inflammation in the kidney (Chinese Pharmacopoeia Committee 2005; Ballabh et al. 2008).

*Viola odorata*, commonly known as Blue Violet, is indigenously found in Iran, Europe and North Africa. Based on reports, infusion of 2 g/animal leaves of *V. odorata* by gastric intubation to rabbits demonstrates a significant diuretic effect (Lim 2014). Furthermore, oral administration of the aqueous extract of this plants aerial parts has shown significant diuretic effects in rats (Vishal et al. 2009). Along with diuretic effects, it has been demonstrated that water-soluble polysaccharides of *V. odorata* can suppress exudation and proliferation phases of inflammation through alterations in capillary permeability (Drozdova & Bubenchikov 2005).

*Echium amoenum* or Borage is a large hairy annual herb mostly found in Northern parts of Iran and different regions of Europe. The flowers and the leaves of this medicinal plant are mostly used in treatment of stress and depression and demonstrate several medicinal properties, most importantly anti-diuretic due to the presence of potassium nitrate and anti-inflammatory effects (Abolhassani 2004).

Along with previous studies, the present study is an evidence-based double blind study in order to evaluate efficiency and safety of mentioned medical plants extracts mixture as an alternative therapy for patients suffering from BPH. During the study, efficacy and safety of plant extracts combination made from *V. odorata*, *E. amoenum* and *P. alkekengi* plants in treatment of BPH in men was examined compared to placebo group.

## Materials and methods

Current randomized, double blind, 2-week placebo-controlled single centre trial was performed to evaluate the efficacy of *V. odorata*, *E. amoenum* and *P. alkekengi* extracts mixture in male patients with BPH. A total of 86 male symptomatic BPH patients were chosen according to inclusion admitted to urology department of Baghiatallah Hospital, Tehran, Iran. Patients were allocated in one of control or placebo group (*n* = 29) and case group (*n* = 57). Demographic data of studied groups are presented in Table 1.

**Table 1.** Patient demographic characteristics.

Parameters	Control (*n* = 29)	Case (*n* = 57)	*p* Value
Mean age (years)	61.62 ± 1.42	60.3 ± 1.13	0.87
High (cm)	172.5 ± 0.94	169.72 ± 1.04	0.68
Weigh (kg)	77.38 ± 1.38	76.31 ± 1.95	0.73
History of BPH (*n*)	17 (58.6%)	21 (36.8%)	0.06
History of smoking (*n*)	14 (48.3%)	23 (40.4%)	0.43
Prostate volume (mL)	42.67 ± 4.34	37.25 ± 2.22	0.22
Urine Flow (mL/s)	7.18 ± 0.9	5.66 ± 0.37	0.33
Extant urine (mL)	55 ± 18.63	45.57 ± 13.05	0.68

### Inclusion criteria

The trial was conducted in accordance to the ethical considerations of the ‘Declaration of Helsinki’ and subsequent amendments thereof (Nuremburg protocol). The committee of ethics at the Baghiatallah University of Medical Sciences (Tehran, Iran) approved the protocol of the study (Reference number: IR.BMSU.REC.1394.246) and written informed consents were collected from subjects before inclusion in the study. Subjects were considered eligible for inclusion in this trial only if all of the following criteria were applicable:

- Male patients with confirmed BPH diagnosis through medical history physical examination including a digital rectal examination (DRE);
- Aged between 40 and 75 years old;
- Prostate volume of more than 30 cm^3^ diagnosed by transrectal ultrasonography (TRUS);
- International Prostate Symptom Score (IPSS) of more than 13 at the screening time;
- Patients which were able and willing to give their written informed consent.

### Exclusion criteria

Patients with diabetes, hypertension, cardiovascular disorders, hyperlipidaemia, history of cardiac apoplexy, cerebral apoplexy, ischemic attack, urinary infectious vessels or prostate and also the ones treated with anti-BPH drugs within a month before the beginning of study or sensitive to applied medicinal plants in the study were excluded from participation.

### Plant material

Plant materials were collected throughout the year 2014 from different zones of Iran. *P. alkekengi* fruits (voucher 975) were collected from Guilan province in September 2014, *E. amoenum* flowers (voucher 976) were collected from a farm at 80 km north of Ghazvin province in March 2014 and *V. odorata* flowers (voucher 977) were collected from Mazandaran forest, Mazandaran, Iran in April 2014. A voucher specimen of each plant was identified by Dr. Abbas Hadjiakhoondi and deposited in Herbarium of Pharmacy School, Tehran University of Medical Sciences Tehran, Iran.

### Preparation of crude extracts

All plant materials were air-dried at room temperature in the shade before extraction. After grinding, 50 g of each dried plant material was mixed and extracted with 80% ethanol by repeated maceration (2 × 48 h). The solvent was completely removed under reduced pressure using a rotary evaporator apparatus. Dried extracts were kept at 4 °C until usage. Final applied formulation in the study was a mixed hydro-alcoholic solution of *P. alkekengi*, *E. amoenum* and *V. odorata* extracts with final concentration of 1.5, 1 and 1.5%, respectively.

### Conduction of the trial

Patients received 1 mL of assigned extract twice daily (12 h intervals and total daily dose of 2 mL) for 2 weeks. Assigning the baseline visit (week 0), patients returned 2 weeks later to urology department for assessing the safety and efficacy of treatment. The value of haemoglobin (Hb), haematocrit (HTC), platelets (PLT), neutrophils, lymphocytes, and basophiles in blood were checked during the trial. Also serum electrolytes, fasting blood sugar (FBS), creatinine, BUN, elements (K) and enzyme activities (AST, ALT and ALP) were measured during the study. Serum examination for measuring prostate specific antigen (PSA) was also performed. IPSS was utilized for evaluating the validity of patient’s symptoms and responses to therapeutic protocol and further comparing the results among control and case groups. Finally, the rate of urinary excretion abnormalities such as nocturia, incomplete urination, frequency, intermittency, urgency and strength of urine flow were also evaluated before and after the therapy. All episodes of adverse effects such as hypertension, nausea, vomiting, dyspepsia, diarrhoea, constipation and rash were also recorded throughout the study.

### Statistical analysis

All data were analysed using Student’s *t*-test or ANOVA with Bonferroni’s adjustment for multiple comparisons. A probability of less than 0.05 was considered as significant. Data were analysed using SPSS, version 16.5 (SPSS Inc., Chicago, IL).

## Results

Assortment of eligible subjects, meeting all inclusion criteria was begun in February 2015 and ended in April 2015. As depicted in Table 1, there were no statistically significant differences between groups regarding profiles and demographic characteristics of patients. The mean age of total participants was 60.74 ± 0.89 years (ranging between 40 and 75 years), and the means of height and weight were 171.55 ± 0.79 cm and 77.01 ± 1.28 kg, respectively. From all 86 subjects, 29 (27%) were included in control group and the others were placed in case groups. No statistically significant differences were observed between groups regarding the mean prostate volume, urine flow rate and extant urine volume.

Table 2 demonstrates the changes in IPSS scores of important urinary excretion abnormalities including nocturia, incomplete urination, frequency, intermittency, urgency and weak flow plus prostate volume and extant urine volume before and after treatment in both control and case studies. Except the Urgency, IPSS score of all mentioned abnormalities in treatment group was significantly decreased after treatment compared to placebo receiving group. Furthermore, the prostate volume was significantly decreased in treatment group in comparison to control group which remained almost intact. Finally, the interesting finding was that the extant urine volume in placebo receiving group was increased after 14 days, in contrast to the observations in treatment group in which mentioned parameter was significantly decreased. The significant difference between the changes in quality of life scores suggests that the patients in treatment group were satisfied with the therapeutic regimen received.

**Table 2.** Comparison of IPSS changes in incidence of important urinary excretion abnormalities.

Changes in incidence of important urinary excretion abnormalities	Placebo receiving group (%)	Treatment group (%)	*p* Value
Incomplete Urination	31.5 ± 1.57	42.3 ± 2.04	0.003
Frequency of Urination less than 2 Hours	8.7 ± 0.43	20.08 ± 1.02	0.001
Intermittency	30.67 ± 1.34	40.78 ± 2.16	0.003
Urgency	61.53 ± 3.21	60.91 ± 3.14	0.82
Weak stream	27.81 ± 1.42	50.58 ± 2.14	0.001
Straining	11.40 ± 0.47	55.67 ± 2.53	0.001
Nocturia	4.6 ± 0.23	40.14 ± 1.89	0.001
Quality of life	21.47 ± 1.07	34.23 ± 1.64	0.001
Overall improvement in IPSS	25.17 ± 1.25	50.84 ± 2.53	0.001
Prostate volume	2.91 ± 0.18^a^	16.92 ± 0.89	0.001
Extant Urine	35.22 ± 3.54^a^	28.12 ± 1.36	0.001

^a^Values were increased.

Table 3 represents the perceived adverse effects in therapeutic plant mixture receiving group including nausea, vomiting, dyspepsia, diarrhoea, constipation, rash and hypersensitivity in comparison with placebo receiving group. No clinically relevant increase or difference in the incidence of adverse events was noticed between the two groups. The most common adverse event in both groups was constipation.

**Table 3.** Adverse effects attributable to the study drug.

Parameters	Control (*n* = 29)	Case (*n* = 57)
Nausea	2	0
Vomiting	1	0
Dyspepsia	0	0
Diarrhoea	1	1
Constipation	9	5
Rash	2	0
Hypersensitivity	1	0

Table 4 demonstrates the data related to the mean variations of biochemical blood test values during the beginning and the end of the trial. No statistically significant differences between the control and treatment groups regarding the mean ranges of transferrin, BUN, Cr, FBS, K, AST, ALT, ALP, Hb, HCT, WBC, PLT, neutrophils and basophiles from baseline to the end of the study were observed.

**Table 4.** The mean value of some biochemical tests before and after study.

Parameters	Control	Cases	*p* Value
BUN			
Before	18.31 ± 1.14	17.86 ± 0.78	0.74
After	16.5 ± 0.66	17.8 ± 1.29	0.51
*p* Value	0.77	0.66	
Cr			
Before	1.17 ± 0.04	1.23 ± 0.03	0.29
After	1.16 ± 0.06	1.26 ± 0.05	0.31
*p* Value	0.43	0.15	
FBS			
Before	118.27 ± 11.8	108.6 ± 4.46	0.42
After	126 ± 11.67	112.25 ± 7.99	0.32
*p* Value	0.70	0.9	
K			
Before	4.19 ± 0.27	19.01 ± 13.29	0.44
After	4.2 ± 0.25	4.6 ± 0.12	0.12
*p* Value	0.35	0.61	
sGOT			
Before	20 ± 1.68	22.68 ± 1.23	0.2
After	22.5 ± 2.68	24.45 ± 2.19	0.59
*p* Value	0.23	0.48	
sGPT			
Before	24.06 ± 2.28	24.43 ± 1.9	0.9
After	27.63 ± 5.37	28.08 ± 2.82	0.93
*p* Value	0.76	0.65	
AlkPh			
Before	201.71 ± 12.4	192.72 ± 20.02	0.74
After	185 ± 16	182.57 ± 10.73	0.91
*p* Value	0.99	0.92	
Hb			
Before	14.61 ± 0.45	17.73 ± 2.61	0.31
After	14.91 ± 0.35	15.46 ± 0.33	0.35
*p* Value	0.82	0.38	
HCT			
Before	44.51 ± 0.62	45.61 ± 0.95	0.47
After	44.57 ± 0.81	44.67 ± 079	0.93
*p* Value	0.80	0.27	
WBC			
Before	6.23 ± 0.3	8.46 ± 1.67	0.43
After	6.16 ± 0.29	10.39 ± 3.28	0.26
*p* Value	0.58	0.56	
Platelet			
Before	206.44 ± 12.25	201.97 ± 6.67	0.72
After	223 ± 13.2	196.38 ± 8.14	0.09
*p* Value	0.86	0.34	
Neutrophils			
Before	28.46 ± 7.05	20.79 ± 6.67	0.37
After	38.48 ± 12.20	25.27 ± 7.66	0.01
*p* Value	0.07	0.26	
Lymphocyte			
Before	29.68 ± 3.17	19.8 ± 2.28	0.01
After	37.04 ± 1.20	23.73 ± 3.11	0.27
*p* Value	0.10	0.18	
Basophiles			
Before	0.00 ± 0.00	0.37 ± 0.35	0.41
After	0 ± 0	0 ± 0	0.43
*p* Value	–	0.39	

Table 5 illustrates the data regarding the mean prevalence of some urinalysis tests such as glucose, protein, ketones, blood, crystals, nitrite, erythrocytes, leukocytes and bacteria in both groups during the study. No statistically significant differences between the control and case groups regarding the urinalysis tests were observed.

**Table 5.** The prevalence of some urinalysis tests during the study.

	Control			Case			
Parameters	Negative	Normal	Positive	Negative	Normal	Positive	*p* Value
Glucose							
Before	8 (32%)	14 (56%)	1 (4%)	14 (27.5%)	35 (68.6%)	1 (2.0%)	0.25
After	1 (12.5%)	6 (75%)	1 (12.5%)	4 (18.2%)	16 (72.7%)	0 (0%)	0.46
*p* Value	0.56	0.56	0.56	0.08	0.08	0.08	
Protein							
Before	23 (92%)	2 (8%)	0 (0%)	49 (98%)	0 (0%)	1 (2%)	0.1
After	8 (100%)	0 (0%)	0 (0%)	22 (100%)	0 (0%)	0 (0%)	–
*p* Value	1.0	1.0	1.0	1.0	1.0	1.0	
Ketones							
Before	24 (100%)	0(0%)	0 (0%)	49 (98%)	0(0%)	1 (2%)	0.4
After	8 (100%)	0 (0%)	0 (0)	22 (100%)	0 (0%)	0 (0%)	
*p* Value	1.0	1.0	1.0	1.0	1.0	1.0	
Blood							
Before	7 (58.3%)	2 (16.7%)	3 (42.5%)	21 (95.5%)	0 (0%)	1 (4.5%)	0.02
After	1 (50%)	0 (0%)	1 (50%)	8 (100%)	0 (0%)	0 (0%)	0.03
*p* Value	1.0	1.0	1.0	1.0	1.0	1.0	
Crystal							
Before	25 (100%)	0 (0%)	0 (0%)	48 (98%)	0 (0%)	1 (2%)	0.47
After	8 (100%)	0 (0%)	0 (0%)	18 (90%)	1 (5%)	1 (5%)	0.65
*p* Value	1.0	1.0	1.0	0.31	0.31	0.31	
Nitrite							
Before	23 (92%)	0 (0%)	2 (8%)	50 (100%)	0 (0%)	0 (0%)	0.04
After	8 (100%)	0 (0%)	0 (0%)	22 (100%)	0 (0%)	0 (0%)	
*p* Value	1.0	1.0	1.0	1.0	1.0	1.0	
Erythrocyte							
Before	17 (70.8%)	0 (0%)	7 (29.2%)	43 (93.5%)	0 (0%)	3 (6.5%)	0.01
After	7 (100%)	0 (0%)	0 (0%)	18 (78.3%)	0 (0%)	5 (21.7%)	0.17
*p* Value	1.0	1.0	1.0	1.0	1.0	1.0	
Leukocyte							
Before	18 (75%)	0 (0%)	6 (25%)	48 (96%)	0 (0%)	2 (4%)	0.01
After	7 (87.5%)	0 (0%)	1 (12.5%)	22 (95.7%)	0 (0%)	1 (4.3%)	0.41
*p* Value	1.0	1.0	1.0	0.56	0.56	0.56	
Bacteria							
Before	18 (78.3%)	0 (0%)	5 (21.7%)	42 (82.4%)	1 (2%)	8 (15.7%)	0.66
After	7 (87.5%)	0 (0%)	1 (12.5%)	21 (95.5%)	0 (0%)	1 (4.5%)	0.44
*p* Value	1.0			0.18			

## Discussion

Several studies have depicted different restrictions in association with BPH therapy by different classes of chemical drugs and invasive procedures including surgery. For instance, administration of Finasteride results in significant decrease in libido and requires a long period of time before it begins its beneficial effects, or α blockers can result chest pain, irregular heartbeats and impotence (Gormley et al. 1992; Debruyne 2000). Consequently, now many clinicians prefer to use natural products to overcome or at least partly improve symptoms associated with BPH. In current single centre, randomized, double blind, placebo controlled study performed on 86 symptomatic BPH patients, we investigated how patients with BPH would respond to the prepared extract of natural products mixture. Interestingly, in patients receiving the mixture of plant extracts, a markedly beneficial response was observed by decreasing in IPSS scores of nocturia, incomplete urination, frequency, intermittency, urgency, weak flow and overall IPSS score compared to placebo receiving group.

In a recently performed study, it was clearly demonstrated that administration of *P. alkekengi* extract significantly reduced testosterone level which may contribute to the decrease in the size of prostate (Naser et al. 2008; Nicholson & Ricke 2011). Furthermore, it has been shown that administration of *P. alkekengi* fruit extract induces a significant antispasmodic effect on uterus of rats mainly through blocking Ca^2+^ and partially via inhibiting NO synthesis and antagonizing β-adrenoceptors (Gharib Naseri et al. 2008).

Several studies have identified the presence of β-sitosterol as an effective component in *V. odorata* (Dweck 2006; Mittal 2013; Lim 2014). β-Sitosterol is considered as a phytopharmacological agent comprising several phytosterols (Berges et al. 1995; Klippel et al. 1997). Based on a report published in Lancet, in a randomized double blind study on 200 symptomatic BPH patients for 6 month receiving either of 20 mg β-sitosterol or placebo, the Boyarsky score was significantly decreased in β-sitosterol receiving group compared to placebo group. Furthermore, the prostate volume was significantly reduced, urine flow rate was increased and the quality of life score, urinary volume retention and mean voiding time were significantly improved. More importantly, no significant adverse effects were observed with β-sitosterol therapy (Berges et al. 1995).

Notably, however, a significant relationship has been reported between depression and anxiety and LUTS in several studies over last few decades (Engel 1964). This association could result from multiple mechanisms. For instance LUTS causes a significant reduction in health related quality of life and end in embarrassment, poor self-esteem, social phobia, anxiety, demoralization and even considered as weakness or sign of aging either by patients themselves or by their partners (Wong et al. 2010; Breyer et al. 2014). Additionally, daytime drowsiness and inability to concentrate are two main consequences of disturbed sleep and nocturia which can further affect patient’s quality of life and development of significant emotional distress (Johnson et al. 2011; Molinuevo & Batista‐Miranda 2012). Interestingly, multiple studies have proposed that administration of *E. amoenum* extract demonstrates several anxiolytic, antidepressant, anti-obsessive compulsive and sedative effects, all of which can significantly improve the quality of life, self-esteem and prolong life expectancy (Sayyah et al. 2006, 2009; Shafaghi et al. 2010).

As mentioned previously, inflammation is another factor mostly involved in BPH etiology and as the extracts of these three plants are all effective anti-inflammatory agents, the other results of this effectiveness is attributed to this point. At the end the diuretic effects of the administered mixture can further improve the urinary flow rate. The other important result obtained in this study was that the urgency was not affected by this mixture which may be related to the diuretic effects of these plants.

Adverse effects, such as nausea, vomiting, dyspepsia, diarrhoea, constipation, rash and hypersensitivity did not differ significantly between groups before and after the trial. Furthermore, the quality of life was improved in treatment group according to patient’s answers.

## Conclusions

Based on results obtained in current study, it can be concluded that administration of mixture of *Viola odorata*, *Echium amoenum* and *Physalis alkekengi* extracts, as naturally occurring compounds, can safely and effectively improve LUTS in symptomatic BPH patients and be considered as a convenient treatment choice for BPH treatment. However, it is difficult to state with certainty whether the same results could be observed through applying merely one or two of the plant extracts presented in the mixture. *P. alkekengi*, *V. odorata* and *E. amoenum* have shown to be successful at least partly, when administered individually. Since each plant possess slightly different mechanisms and time frame of action, it appeared more logical to evaluate the combination of extracts to identify clinical utility first. At the end, further studies for comparing the therapeutic outcomes of each component individually with mixture and investigating whether extending duration of therapy could further affect the final outcomes seems to be essential.
